# ‘Unless you come with your partner you will be sent back home’: strategies used to promote male involvement in antenatal care in Southern Tanzania

**DOI:** 10.1080/16549716.2018.1449724

**Published:** 2018-04-27

**Authors:** Apollonia Kasege Peneza, Stephen Oswald Maluka

**Affiliations:** a Masasi Town Council, Masasi, Tanzania; b Institute of Development Studies, University of Dar es Salaam, Dar es Salaam, Tanzania

**Keywords:** Male involvement strategies, antenatal care, pregnancy and childbirth, Tanzania

## Abstract

**Background**: Male involvement in pregnancy and childbirth has been shown to improve maternal and child health. Many countries have used different strategies to promote participation of men in antenatal care services. While many strategies have been employed to promote male participation in antenatal care, few have been evaluated to provide much-needed lessons to support wider adoption.

**Objective**: This study aimed at describing strategies that were used by health providers and the community to promote male participation in antenatal care services and challenges associated with the implementation of these interventions in Southern Tanzania.

**Methods**: We used qualitative data and analytical methods to answer the research questions. The study relied on semi-structured interviews with health providers, men and women, village and community leaders and traditional birth attendants. Data were analysed using a thematic approach.

**Results**: The findings of this study revealed that different strategies were employed by health providers and the community in promoting participation of men in antenatal care services. These strategies included: health providers denying services to women attending antenatal care without their partners, fast-tracking service to men attending antenatal care with their partners, and providing education and community sensitisation. The implementation of these strategies was reported to have both positive and unintended consequences.

**Conclusions**: This study concludes that despite the importance of male involvement in pregnancy and childbirth-related services, the use and promotion of the male escort policy should not inadvertently affect access to antenatal care services by pregnant women. In addition, programmes aiming for men’s involvement should be implemented in ways that respect, promote and facilitate women’s choices and autonomy and ensure their safety. Furthermore, there is a need for sensitisation of health providers and policymakers on what works best for involving men in pregnancy and childbirth.

## Background

In many low- and middle-income countries, men, being the principal breadwinners and key decision-makers in the household, normally influence women’s access to maternal and child health services [1–3]. The potential influence of men on maternal and child health can be better conceptualized through the three delays model leading to maternal mortality [4]. The first two delays at the community level – namely, the delay in decision-making on healthcare-seeking, and the delay in reaching the health facility – are among factors influencing husbands’ attitudes and healthcare-seeking practices [4].

The most common strategies which have been promoted to invite the support and involvement of men in pregnancy and childbirth include mass media campaigns, workplace and community outreach and health education for men and women, and facility-based counselling for couples [5]. Studies have documented the benefits of involving men in pregnancy and childbirth, including increased access to antenatal care (ANC) services and thereby the increased likelihood of delivery by skilled birth attendants [6]; increased health education for both women and men [7,8]; increased likelihood of using modern family planning methods [9,10]; and addressing gender-related barriers to access to maternal and child health services [11,12].

However, the World Health Organization (WHO) recommends that interventions to promote male involvement in maternal and child health should be implemented provided that they respect, promote and facilitate women’s choices and their autonomy in decision-making [5]. This implies that women should be consulted and their opinions should be taken into account when designing interventions to promote male involvement in pregnancy and childbirth. This will help overcome potential harms related to male involvement in maternal and child health.

Despite various efforts to promote male involvement, in many low- and middle-income countries, male participation in pregnancy and childbirth matters is disappointingly low [13–16]. For example, a Ugandan study reported that the majority (74%) of respondents had a low male involvement index and only 5% of men escorted their partners to the ANC clinic [13]. Similarly, several studies conducted in Tanzania have reported a low rate of male participation in prevention of mother-to-child transmission (PMTCT) and in voluntary counselling and testing [15–17].

Studies have reported several barriers to male participation in pregnancy and childbirth. The common barriers include: socially constructed gender roles, lack of knowledge of maternal health matters [13,14,16–19], and health-system-related barriers such as low quality of services and negative attitudes of the health providers [16,17,19–23].

In Tanzania, maternal and child health policies, guidelines and strategies recognise the importance of engaging men in reproductive and child health. For example, focused antenatal care (FANC) guidelines encourage couples to attend ANC clinics together [24]. Similarly, the PMTCT guidelines encourage participation of men in voluntary counselling and testing (VCT) [25]. Pregnant women are, therefore, required to come with their partners during the first ANC attendance for VCT [24,25].

Similarly, health service providers in Tanzania have been implementing different strategies to promote male involvement in pregnancy and childbirth. Some health service providers offer incentives to couples who attend ANC clinics together. Similarly, some non-governmental organisations (NGOs) and health facilities also organise sensitisation and education programmes for couples to prepare them for pregnancy care and childbirth. In other areas, pregnant women attending ANC without partners receive systematic coaching from health providers on how to request male partners to attend ANC clinics [26].

While many strategies have been used to promote male participation in pregnancy and childbirth, few have been evaluated to provide much-needed lessons for scale-up. More importantly, there is paucity of studies on the implementation of locally based strategies to encourage male partners’ participation in pregnancy and childbirth services. This paper describes strategies that were used by the health providers and the community as an attempt to promote male participation in antenatal care services and challenges associated with the implementation of these interventions in Southern Tanzania.

## Methods

### Study settings

The study was carried out in Masasi District Council in the Mtwara Region. The district had a total population of 260,856 which comprised 125,151 males and 135,705 females [27]. The district was purposively selected because the first author had an active role in the district. In Tanzania, 63% of women give birth in health facilities although over 90% of pregnant women attend ANC for at least one visit [28]. In addition, only 51% of pregnant women complete the recommended four ANC visits and only 24% start ANC attendance before the fourth month of pregnancy [28]. The pattern in rural areas of Tanzania is almost similar. The study involved one hospital, one health centre and four dispensaries. These health facilities were selected in collaboration between the authors and the district health management. The criteria for selecting these health facilities included availability of maternal and child health services as well as geographical accessibility to the health facilities and their catchment villages.

### Study design

This study adopted descriptive qualitative case study design. Descriptive case study design was considered relevant because the study aimed to describe strategies used by the health providers and the community to promote male involvement in pregnancy and childbirth and the real-life context in which they were implemented [29]. The study relied on semi-structured interviews with key respondents. The first author had a specific role in the community and therefore data were collected by a research assistant from April to June 2016. Respondents were purposively selected and included currently pregnant women attending ANC, women who had given birth in the past 12 months, health providers responsible for reproductive and child health at the selected health facilities, village and community leaders, social workers and traditional birth attendants. Women and health workers were recruited at the district hospital, one health centre, and four dispensaries. Health facilities were randomly selected. Interviews were carried out in the respective health facilities where women were found. Semi-structured interview guides were developed for each category of respondents (see additional files). Interviews lasted between 20 and 30 minutes and were audio-recorded with the permission of the respondents. As indicated in Table 1, a total of 53 interviews were carried out.

**Table 1.** Category of respondents.

S/N	Category of respondent	Number
1	Pregnant women	8
2	Women who had delivered within the last 12 months	12
3	Male partners	12
4	Health providers	6
5	Traditional birth attendants	3
6	Religious leaders	5
7	Village leaders	5
8	CSO/CBO/NGO	1
9	District health managers	1
	**Total**	**53**

### Data processing and analysis

Data were analysed using a thematic approach proposed by Braun & Clarke [30]. The following iterative process was followed in data analysis. First, recordings of the interviews were transcribed verbatim in Kiswahili by AKP and checked for accuracy by SM. Only selected quotes used in the study were translated from Kiswahili to English. Second, both authors went through the data in order to understand the depth and breadth of the data-set. Third, AKP developed a list of initial codes based on the objectives of the study. Interviews were then coded manually based on these initial codes. Other codes which emerged during coding were added along the way. Fourth, we sorted the different codes into potential themes by looking into patterns in the coded data. Finally, themes were refined by reading and rereading the data-set. The main overarching themes that were finally identified were: health providers denying services to women attending attending antenatal care without partners, fast-tracking services to men attending antenatal care with their partners, and giving education and community sensitisation.

## Results

The key findings of the study are organised into three broad themes, namely: health providers denying services to women attending antenatal care without partners, fast-tracking services to men attending antenatal care with their partners, and providing education and community sensitisation.

### Health providers denying services to women attending ANC without partners

In Masasi District, pregnant women were required to attend the first antenatal care clinic with their partners or else they would be denied services. This was mainly aimed at achieving couple voluntary counselling and testing as a key component of the PMTCT programme. In case a pregnant woman had an important reason for not going with her partner, such a woman was required to get a letter from village leaders exempting her from bringing a partner to hospital. One of the health workers exemplified it this way:

If a woman comes here the first day without her husband, we ask her to go back home to bring her husband. The village government helps to testify that the woman has no husband or to prove whether a husband has a problem that prevents him from coming to the hospital. (Health worker, Mpindimbi dispensary)

A similar view was reported by almost all the women and men who were involved in this study. One woman reported it this way:

Unless you come with your partner you will be sent back home, you must come with a partner. Otherwise, come with a letter from the Village Executive Officer. (A woman respondent from Mpindimbi village)

This finding was also supported by all the village leaders who were interviewed. One village leader illustrated it this way:

We work together with health service providers in the implementation of the strategy which requires pregnant women to go to hospital with their partners during the first visit. For those who do not have partners must go with a letter from village leaders. I have written a number of letters to introduce pregnant women who do not have partners to the health service providers. (Village chairperson)

The implementation of this strategy was found to have both advantages and disadvantages. The advantage of this strategy was that it forced men to go with their wives as they attended antenatal care on the first visit. It was evident that men feared that their partners would be denied services if they did not go with them. According to our respondents, the implementation of this strategy succeeded in encouraging men to escort their partners for ANC, particularly on the first visits. The quotation below exemplifies this point:

The strategy has motivated men to attend clinics with their wives especially on the first visit. For example, this month we received five women. All of them were accompanied by their husbands. All of them took HIV test and were provided with education on birth preparedness. (Health worker, Mpindimbi dispensary)

However, some respondents reported that in some villages the implementation of this strategy increased the problem of late booking (starting antenatal clinic within 12 weeks as required by the ANC guideline). Some men feared to go to the health facility because they would be required to undertake an HIV test. According to our respondents, some pregnant women delayed starting antenatal clinic because of lack of partner’s support. One of the health service providers made the following remarks:

The strategy of forcing men to come to hospital has made some women to start antenatal care clinics late due to clashes with their husbands about HIV testing. There are cases when a woman comes to register for antenatal care in the sixth or seventh month of pregnancy. They come after they discover that they have no option. (Health worker, Chiungutwa dispensary)

Another health service provider added this:

The strategy of sending pregnant women back home has some challenges. Yesterday we were in a meeting to deliberate on quality improvement where we were told that this strategy has both positive and negative effects. It makes some women not to attend clinics. We have been told not to send them back home but rather to educate them. (Health worker, Mkomaindo hospital)

The findings of the study further indicated that in some villages male partners colluded with a few unethical village leaders so that they could write fake letters to enable women to receive antenatal care services at the health facilities. Likewise, some pregnant women produced false statements either to the village leaders or to the health service providers so that they could attend without their partners. Also, there was a concern among village leaders that the strategy was not legally recognised. There were no by-laws at the village or district level to support implementation of this strategy. As a result, in some of the villages, some village government leaders provided little support in the implementation of the strategy. One of the village government leaders made the following remarks:

As a matter of fact, the village has no by-law that requires a husband to accompany a wife while attending a clinic at a dispensary. This strategy is implemented by the health providers and we are supporting them to implement the strategy. (Village government leader, Nangoo village)

Another leader exemplified it this way:

This strategy which is meant to make women go for check-up with their husbands is not established legally. As a matter of principle, health facilities do not have power to prepare by-laws. Local by-laws are supposed to be initiated by the village leaders (the government). This would make the strategy more powerful. (Village government leader, Mpindimbi village)

### Fast-tracking service to men attending antenatal care with their partners

The second strategy which was employed by the health care providers to promote male participation in antenatal care was giving priority to women who were accompanied by their male partners or to men who brought their children for routine postnatal services. The health providers encouraged women to come with their husbands and partners so that they were assured of fast-tracking services. The majority of health providers had the opinion that men were the main family breadwinners and thus they had to find a balance between attending clinics and income-generating activities. It was evident that this strategy was practised in all facilities which were involved in this study. One of the women respondents at Mkomaindo district hospital made the following remarks:

In this facility we have a strategy to promote male involvement. If a woman comes for antenatal care services with her husband, they are attended first. This is meant to motivate men who attend clinics with their partners. (Female respondent, Mkomaindo hospital)

Another respondent exemplified it this way:

I am happy nowadays [that] at our dispensary men who go for antenatal clinics with their partners are attended first. So, men do not stay in a long queue waiting for health services. This is an incentive for the couples and the practice should be maintained. (Interview with a male partner)

Unlike the previous strategy, the fast-tracking services strategy did not face technical challenges. This initiative also seemed to be preferred by women because they spend little time at the health facilities. However, while this strategy succeeded in attracting men to accompany their partners to the health facilities, the majority of men did not escort their partners, mainly due to fear of undertaking an HIV test. The vast majority of respondents reported that had it not been for the HIV test, the majority of men would have accompanied their partners for antenatal visits. One respondent illustrated it this way:

[The] majority of men do not go with their partners for antenatal care because they are afraid of HIV testing. This is a big challenge for many male partners. (Interview with a pregnant woman, Chihungutwa dispensary)

### Education and community sensitisation

Another strategy which was employed in Masasi District to promote male participation in pregnancy and childbirth was educating and sensitising communities on the importance of involving men in pregnancy and childbirth. The main objective of this strategy was to educate men and the community at large on the importance of the male partner’s participation in maternal and child health-related matters. The main channels which were used included public meetings, community outreach activities, radio and mobile phone messages. Education was also provided as part of the routine ANC services. During village meetings, health workers were invited and given the chance to speak to the community on various matters related to pregnancy and childbirth:

During ANC, we normally provide health education, including the importance of male involvement in maternal and child health. We also get opportunities to sensitise community members during village meetings and social gatherings. (Interview with a health provider)

Sensitisation and health education in the communities were also provided by the community health workers. One respondent reported it this way:

Our village has two community health workers who provide health education, including issues related to pregnancy and childbirth. During community meetings, they also get [the] chance to speak with the community. (Interview with a male partner, Chihungutwa village)

In some cases, health providers requested religious leaders to communicate important information on maternal and child health matters during religious gatherings. Religious leaders were also occasionally invited to attend meetings convened by the health providers in the health facilities. Further, in some villages there were pilot projects which provided important maternal and child health information to the communities using short messages via mobile phones. Community members could send questions and get responses instantly.

## Discussions

This study aimed at assessing strategies that were used by health providers and the community to promote male participation in antenatal care services, and challenges associated with the implementation of these interventions in Southern Tanzania. The most common strategies were: health providers denying services to women attending antenatal care without partners, fast-tracking men attending antenatal care with their partners, and education and community sensitisation. The remaining section discusses these strategies in light of other studies on male involvement in ANC in low- and middle-income countries.

### Health providers refusing services to women attending ANC without partners

The most widespread and popular strategy used by health providers to promote male participation in ANC in Southern Tanzania was refusing services to women attending ANC without partners. A similar finding has been reported in recent studies in Tanzania and Malawi [31,32]. These studies indicated that pregnant women who did not bring their partners were denied ANC services. Our findings indicated that while this strategy succeeded in forcing men to accompany their wives, it increased the risks of late initiation of antenatal care attendance. Some men feared going to the health facility because they would be required to test for HIV. As a result, some pregnant women delayed starting ANC clinic due to lack of partner’s support. A recent Tanzanian study reported that the requirement to bring one’s spouse resulted in a delay in the initiation of the ANC and in some cases pregnant women skipped the ANC attendance [31].

While attending ANC with a husband is important for the health of pregnant woman and the newborn, this strategy increases the burden on women to convince their husbands to go with them to the health facilities. In a society where cultural gender norms have for a long time excluded men from maternal and child health responsibilities, it is unfair to put more pressure on women to come with their partners. In addition, this strategy infringes a woman’s rights. From a rights-based perspective, pregnant women have a right to access health services without being turned back.

The district health managers need to explore other strategies for male engagement that will promote the participation of men in pregnancy and childbirth services without affecting women’s rights to access maternal and child health care. For example, women who do not come with their partners could be given services and requested to come with their partner during the following visit. In Malawi, while an HIV test was part of the routine ANC, when a woman started ANC attendance she was requested to come with her husband during the next visit [33]. In Tanzania, coaching of pregnant women on how to invite their partners, followed by written invitations, increased the number of women who attended ANC with their partners [26]. Invitation letters have been widely used to promote male participation in PMTCT in various settings [26,33–36]. Invitations should highlight the importance of ANC visits for men.

Furthermore, the district health authorities should ensure that health facilities provide a male-friendly environment and services that target men. This would help increase the benefit of male involvement and reduce harm to pregnant women. For example, extending the hours for providing services, such as weekend clinic hours and earlier morning hours, and shorter clinic waiting times could encourage men’s participation in antenatal care services [13,18].

### Fast-tracking men attending antenatal care with their partners

Providing fast-tracking services was another strategy which was widely used to promote male participation in Southern Tanzania. A similar strategy was reported in Malawi and Kenya. For example, in Malawi, women who attended ANC clinics together with their husbands were given priority in receiving health services [33,37]. In Kenya, health service providers promoted male involvement in ANC through fast-tracking men attending with their partners and giving a free shawl for their child [38]. The implementation of this strategy was based on the assumption that men are the breadwinners and thus do not want to spend hours waiting for health services. This would affect income-generating activities. Long waiting times at the health facility have been reported to deter the participation of men in maternal and child health services [13,21]. These studies have reported that in most of the health facilities in low- and middle-income countries, women spend a long time waiting for the ANC services, mainly due to a shortage of health providers. Men who are employed are often discouraged if they have to wait for a long time before receiving ANC services [13]. While this strategy managed to attract men to attend ANC clinics together with their wives, it may not be sustainable in the long term when the number of men who accompany their partners increases.

Providing favours for couples who attend ANC together can result in stigmatisation of women who do not have steady partners and husbands [33]. Furthermore, this requirement might pose a challenge to women’s autonomy in cases when women might have a male partner but for diverse reasons, such as intimate partner violence or controlling behaviour, might not want him to be involved. This finding reinforces the WHO recommendations that interventions aimed to promote male involvement in maternal and child health should be implemented provided that they respect, promote and facilitate women’s choices and their autonomy in decision-making and ensure their safety [5]. Women should be consulted and their opinions should be taken into account when designing interventions to promote male involvement in pregnancy and childbirth. This will help overcome potential harms related to male involvement in maternal and child health.

### Health education and community sensitisation

Health education and community sensitisation campaigns were another strategy which was used by the health providers and village leaders to promote male participation in pregnancy and childbirth. Several studies have already documented the effectiveness of education and sensitisation campaigns in promoting effective change in beliefs and behaviour [39–42]. Education interventions which have targeted women and men have been reported to increase knowledge and health-seeking behaviour and to raise awareness on issues related to maternal and child health [39–42]. The main challenge is that, most often, sensitisation campaigns are short-lived and may not produce long-term sustainable behaviour change [33]. In addition, community mobilisation campaigns require a massive mobilisation of human and financial resources, which may not always be available. In order to tackle this challenge, the health providers in collaboration with the district health managers should empower community health workers to provide education and community sensitisation campaigns in their respective villages, particularly during political and public meetings, and other social gatherings. In addition, religious leaders should be sensitised and empowered to provide education to the general community, and men in particular, during religious gatherings. Working with religious and traditional leaders can be very necessary due to their influence in the community [18,33].

## Strengths and limitations of the study

The key strength of our study is that analysis was based on the data generated from multiple respondents including health providers, male partners, pregnant women, and village and traditional leaders. This provided a good opportunity to triangulate the findings across different categories of respondents. Notwithstanding this strength, the study was carried out in one district and the findings may not sufficiently reflect the realities of other districts in Tanzania. In addition, the first author had an active role in the district. Although this author was not involved in the data collection, the author’s bias might have influenced the analysis.

## Conclusion

This study has reported various strategies which were used to promote the participation of men in antenatal care in Southern Tanzania and the associated challenges. While these strategies are intended to encourage men to escort their wives during ANC visits, they put more pressure on women to convince their partners in a culture where this has been considered as a woman’s domain. This study concludes that despite the importance of male involvement in pregnancy and childbirth-related services, health providers and the district heath authorities should ensure that use and promotion of the approach does not inadvertently affect access to antenatal care services by pregnant women. In addition, interventions aimed to promote male involvement in maternal and child health should respect, promote and facilitate women’s choices and their autonomy in decision-making and ensure their safety. Women should be consulted and their opinions should be taken into account when designing interventions to promote male involvement in pregnancy and childbirth. Furthermore, there is a need for sensitisation of health providers and policymakers on what works best for involving men in pregnancy and childbirth.

## Paper context

While many strategies have been implemented to promote male participation in antenatal care, few have been evaluated to provide much-needed lessons to support wider adoption. This paper describes strategies that were used to promote male participation in antenatal care in Tanzania. There is a need to explore other strategies for male engagement that will promote the participation of men in pregnancy and childbirth services without affecting women’s rights to access maternal and child health care.
